# Chromophobe renal cell carcinoma with nodal involvement: A rare case report highlighting unusual molecular features

**DOI:** 10.1016/j.eucr.2026.103527

**Published:** 2026-07-01

**Authors:** Sam Kwon, Tali Newman, Fiona Wardrop, Katherine Zhong, Josiah Dallmer, Andrew Hall, Michael Whalen

**Affiliations:** a The George Washington University School of Medicine and Health Sciences, Washington, DC, USA; b Department of Pathology, The George Washington University School of Medicine and Health Sciences, Washington, DC, USA; c Department of Urology, The George Washington University School of Medicine and Health Sciences, Washington, DC, USA

**Keywords:** Chromophobe renal cell carcinoma, Neoplasm metastasis, Sarcomatoid renal cell carcinoma, Mutation

## Abstract

Chromophobe renal cell carcinoma (ChRCC) is a renal malignancy typically associated with indolent behavior, with nodal involvement being rare. We report a 78-year-old African American male with incidentally discovered node-positive (pN+) ChRCC with sarcomatoid differentiation who developed spinal metastases despite adjuvant pembrolizumab. Histopathologic evaluation demonstrated distinct plant-like cell membranes, while genomic profiling revealed CDK4 amplification and MTAP and CDKN2A alterations associated with aggressive tumor biology. Notably, the tumor also exhibited additional unique molecular alterations that have not been previously reported in the literature.

## 1 Introduction

Chromophobe renal cell carcinoma (ChRCC) is the third most common subtype of renal cell carcinoma (RCC) and typically confers an excellent prognosis compared to other primary renal cell carcinoma subtypes.^1^ This favorable prognosis reflects the indolent nature of ChRCC. Most tumors are detected incidentally on abdominal imaging at an early stage and rarely present with lymphovascular invasion. This indolence is attributed to a low somatic mutational burden with TP53 and PTEN being the only recurrently mutated genetic markers, a tumor microenvironment with low tumor specific T-cell expansion, and low immune checkpoint expression^2^^,^^3^.

A small but clinically significant portion of ChRCC published cases from 2011 to 2025 exhibit aggressive behavior with poor outcomes. One recognized adverse feature is sarcomatoid transformation. The largest dedicated pathologic series of sarcomatoid ChRCC (n = 22) reported distant metastases in 73% of patients with a median survival of less than 1 year after diagnosis.^4^ Aggressive ChRCC has also been found to display not only the typical TP53 and PTEN mutations, but also RB1 mutations, imbalances chromosome duplication, and mTOR pathway alterations.^5^

This case highlights the atypical molecular features associated with aggressive ChRCC and contributes to the limited literature characterizing this rare clinical phenotype.

## 2 Case presentation

We report the case of a 78-year-old African American male who was found to have an 8.8-cm left renal mass incidentally identified on computed tomography (CT) of the abdomen and pelvis. His medical history was notable for hypertension, hyperlipidemia, type 2 diabetes mellitus, osteoarthritis, peripheral neuropathy, acute right shoulder bursitis, left eye retinal vein occlusion, and prior pT2 prostate cancer s/p retropubic radical prostatectomy (RRP) without evidence of biochemical recurrence with 29 years follow-up. His surgical history included RRP and left shoulder rotator cuff and tendon repair.

**Fig. 1:**
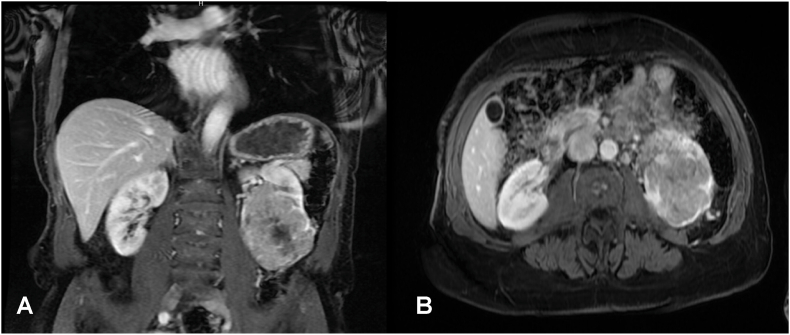
Computed tomography (CT) imaging A. CT abdomen/pelvis demonstrating an 8.8 × 6.3 cm exophytic mass arising from the lower pole of the unenhanced left kidney - coronal view B. Left renal mass with subcentimeter retroperitoneal lymphadenopathy - axial view.

The patient initially presented with a left groin bulge, prompting further evaluation with magnetic resonance imaging (MRI) of the abdomen, which demonstrated a 9.3-cm predominantly exophytic, heterogeneously enhancing, T1-isointense infiltrative mass arising from the left kidney, along with subcentimeter retroperitoneal lymphadenopathy. Subsequent non-contrast CT of the abdomen and pelvis confirmed an exophytic mass arising from the lower pole of the unenhanced left kidney measuring 8.8 × 6.3 cm, as well as an 8-mm para-aortic lymph node (Fig. 1). Scrotal ultrasound additionally revealed a grade 3 left varicocele.

The patient elected to proceed with left robotic-assisted radical nephrectomy, left robotic retroperitoneal lymph node dissection, and left robotic varicocelectomy. His postoperative course was uncomplicated, and he was discharged on postoperative day 3. Final surgical pathology demonstrated chromophobe ChRCC with sarcomatoid features, staged as pT3aN1 (1/8 lymph nodes positive) Mx R0, with lymphovascular invasion involving a segmental branch of the renal vein and renal sinus fat, consistent with AJCC 8th edition stage III disease.

**Fig. 2:**
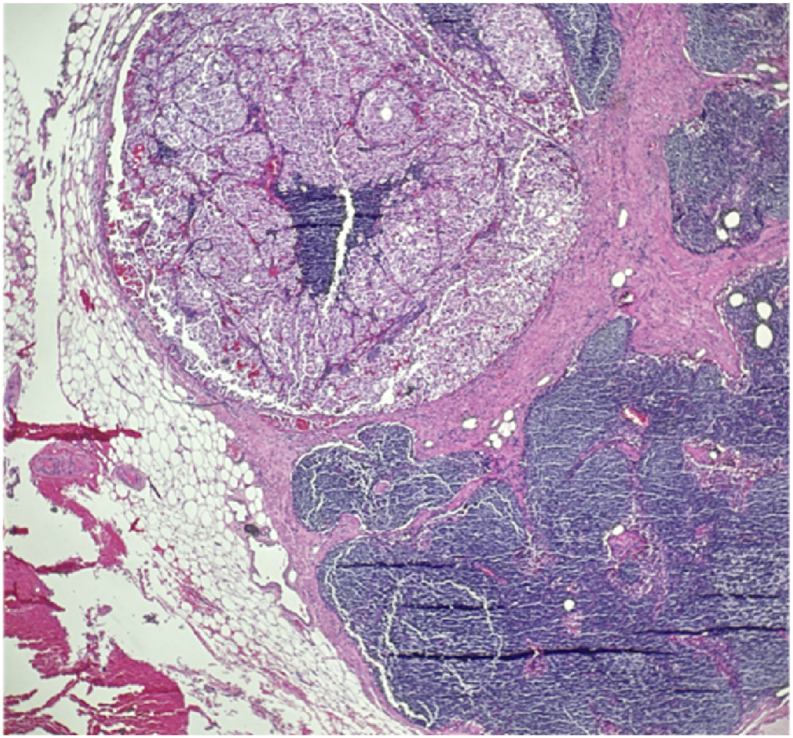
Hematoxylin and eosin (H&E) staining of the chromophobe renal cell carcinoma in a lymph node.

**Fig. 3:**
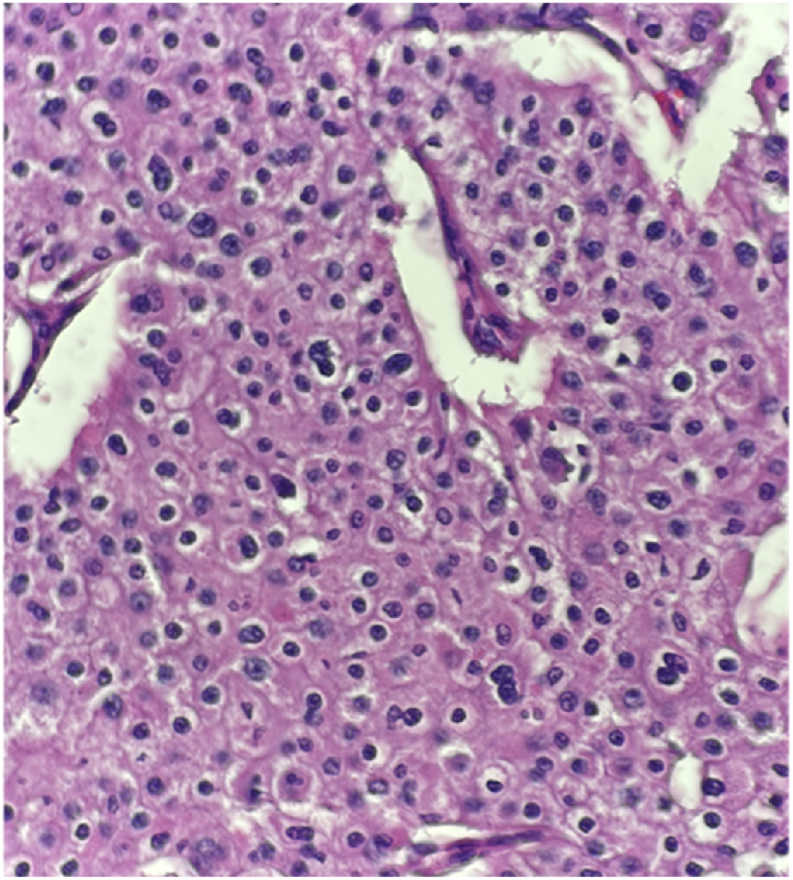
Hematoxylin and eosin (H&E) staining demonstrating typical histologic features of chromophobe renal cell carcinoma, including raisinoid cytoplasm, binucleation, and prominent plant-like cell membranes.

**Fig. 4:**
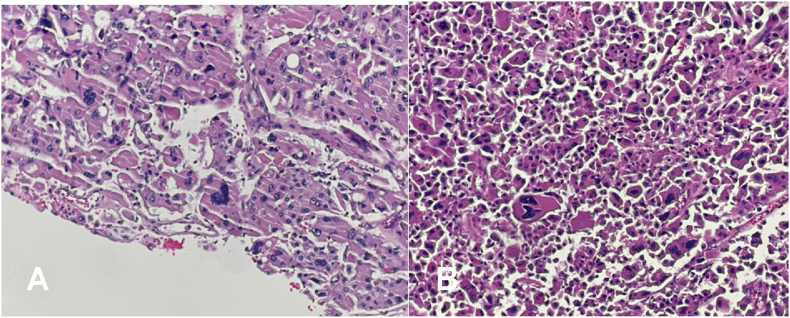
Hematoxylin and eosin (H&E) staining A. Showing sarcomatoid areas with marked cytologic atypia in the nephrectomy specimen. B. Another sarcomatoid tumor area demonstrating similar cytologic atypia.

Histologic examination of the lymph node showed involvement by chromophobe renal cell carcinoma (Fig. 2) and characteristic features chromophobe renal cell carcinoma including raisinoid nuclei, binucleation, and distinct plant-like cell membranes (Fig. 3) were identified. Sections from the nephrectomy showed foci of sarcomatoid differentiation with marked cytologic atypia (Fig. 4). PD-L1 combined positive score (CPS) was 20-30.

Postoperatively, the patient completed 10 cycles of adjuvant pembrolizumab given the sarcomatoid features along with PD-L1 positive staining. The patient experienced side effects of diarrhea, with colonoscopy revealing small intestinal bacterial overgrowth (SIBO) and campylobacter treated with antibiotics. Routine surveillance imaging at 9 months post-surgery with MRI abdomen revealed no local recurrence in the renal fossa, but new mass-like area of enhancing signal alteration involving the posterior elements right of the midline at L1-L2 of the lumbar spine, measuring 17 × 20 mm. Subsequent dedicated cervical, thoracic, and lumbar spine MRI revealed multiple metastases: 3cm metastatic lesion involving the right C7-T1 posterior elements and narrowing the right neuroforamen; 1.8 cm enhancing mass in the right L1 lamina/spinous process. The patient underwent IR-guided biopsy of the cervical/thoracic paraspinal mass which revealed metastatic renal cell carcinoma (CK7 and PAX8 positive, CD117 focally positive, no sarcomatoid features seen). The biopsy was complicated by right hand weakness and peripheral neuropathy. The patient was started on IV cabozantinib and underwent stereotactic body radiotherapy (SBRT) to the involved spinal mets. At this time, post-radiation imaging is still pending.

## 3 Discussion

Chromophobe renal cell carcinoma is the third most common subtype of non-clear cell renal cell carcinoma^1^. Most patients with ChRCC present incidentally with asymptomatic renal masses, similarly to other RCC subtypes, though some may report flank pain, hematuria, or a palpable mass. There are no pathognomonic features for ChRCC on cross-sectional imaging, making differentiating between benign renal oncocytoma (RO) challenging.^6^ Features of sarcomatoid, glandular, or anaplastic dedifferentiation are associated with a worse prognosis.^7^ The etiology of ChRCC is not fully understood, but smoking, obesity, arterial hypertension, and family history are recognized risk factors. When feasible, partial and radical nephrectomy is the gold-standard of curative therapy for ChRCC. In metastatic disease, ChRCC tends to be highly resistant to chemotherapy, while immunotherapy and targeted molecular therapy have recently been shown to be more effective.^8^

Although typically an indolent presentation, a subset of ChRCC exhibits metastatic potential and sarcomatoid dedifferentiation, with limited understanding of the underlying risk factors and genetic drivers. Here, we report a case of ChRCC that presented with lymph node invasion and sarcomatoid features, incorporating molecular profiling and histopathologic findings to characterize genetic alterations associated with more aggressive disease.

The patient's tumor displayed the typical histopathologic features of ChRCC such as raisinoid nuclei, binucleation, and prominent plan-like (thick, eosinophilic) cell membranes. However, abnormal sarcomatoid areas are also identified and characterized by malignant spindle cells reminiscent of pleomorphic undifferentiated sarcoma with marked cytologic atypia. This case fits within the description of other sarcomatoid ChRCC cases.

Analysis of the TEMPUS 648 gene molecular profiling assay (Tempus AI, Inc., Chicago, IL) demonstrated overexpression of EZH2, TOP2A, CDK4, and NY-ESO-1, along with underexpression of CDKN2B mRNA. Similar to findings by Baraban et al., which identified RB1 mutation as a defining molecular feature of high-grade chromophobe RCC (ChRCC), our tumor exhibited overexpression of CDK4, a key upstream kinase regulating the RB1 protein.^9^ Although CDK4 overexpression and RB1 mutation disrupt the same RB pathway, they do so via distinct mechanisms. This suggests that CDK4 overexpression may represent an alternative molecular alteration contributing to high-grade ChRCC. To our knowledge, prior studies have not specifically evaluated EZH2 and TOP2A mRNA expression in ChRCC. Overexpression of these genes provides a biologically plausible explanation for the aggressive behavior observed in our case, potentially through epigenetic silencing of tumor suppressor genes (via EZH2) and promotion of cellular proliferation (via TOP2A). Demirović et al. reported that NY-ESO-1 expression is uncommon in typical ChRCC.^10^ Therefore, its overexpression in our tumor may represent an additional molecular pathway associated with aggressive tumor behavior and highlights a potential avenue for future investigation.

Further analysis using the TEMPUS xT Companion Diagnostic assay identified mutations in TP53, KMT2D, TSC1, CDKN2A, CDKN2B, and MTAP. In line with Alaghehbandan et al., CDKN gene alterations are among the most frequent mutations in ChRCC, although they are not specific to aggressive disease.^11^ Notably, the co-occurrence of MTAP and CDKN2A alterations has been shown by Xu et al.^12^ to be associated with sarcomatoid differentiation and poor clinical outcomes, which is consistent with the features observed in our tumor. Moreover, multiple studies have identified TP53 mutation as a key driver of high-grade and sarcomatoid ChRCC, which was also found in our patient's tumor^9^^,^^13^. The identification of a TSC1 mutation in our case parallels the TSC2 alterations reported by Ahn et al., although these alterations are not specific to aggressive disease^13^. Similarly, KMT2D mutations observed in our tumor are consistent with findings by Gobel et al., but are likewise not specifically associated with aggressive ChRCC.^14^

The PD-L1 immunohistochemistry testing performed at the patient's treating institution demonstrated a combined positive score. This finding is consistent with prior reports of frequent PD-L1 upregulation in sarcomatoid ChRCC^4^^,^^13^, suggesting that PD-1/PD-L1-mediated immune suppression may contribute to the aggressive biologic behavior observed in this case. Nevertheless, the immunogenic microenvironment and therapeutic implications of immunotherapy in aggressive ChRCC remain poorly defined and warrant further investigation.

The majority of patients with ChRCC present with organ-confined disease and lymph nodes and distant metastases are rare. Metastatic chRCC is highly resistant to chemotherapeutic agents, therefore, treatments with various immunotherapies are currently preferred. Systemic therapies include cabozantinib, cabozantinib + nivolumab, and levatinib + pembrolizumab; however, these recommendations are not chromophobe-specific. Early landmark trials such as the TARGET trial and RECORD-1 trial evaluated targeted therapies such as sorafenib and everolimus in metastatic RCC populations, including a subset of patients with chromophobe histology^15^^,^^16^. These patients showed a modest response, helping establish VEGF and mTOR pathway inhibition as new approaches to treatment.

The SUNNIFORECAST was the first clinical trial to investigate combination therapy with nivolumab and ipilimumab compared to sunitinib monotherapy as first-line therapy in patients with unresectable/metastatic non-clear cell RCC. Ipilimumab/nivolumab demonstrated a significantly longer overall survival at 12 months with an acceptable toxicity profile ^17^. KEYNOTE-427 was a Phase 2, single arm trial which assessed the efficacy and safety of pembrolizumab, a PD-1 inhibitor, in advanced nccRCC. Among 21 patients with chromophobe histology, the objective response rate (ORR) was 9.5%, which was significantly lower than other subtypes^18^.

Current literature remains relatively sparse for chromophobe specific treatment of RCC. Although ChRCC is generally associated with a favorable prognosis, the determinants of aggressive behavior in this subtype remain poorly defined. Key steps in the molecular pathway of sarcomatoid transformation have been described via the p53 and RB1 mutations, yet much remains unknown regarding other potential molecular markers such as EZH2, TOP2A, NY-ESO-1, and PD-1/PD-L1–driven immune suppression. Further future investigation into these alterations and their subsequent clinical significance in routine pathologic testing are warranted.

Perhaps the most significant gap in ChRCC care is the lack of a defined standard of care for metastatic disease. Current NCCN guidelines group ChRCC within the broader category of non-clear cell RCC without any histological-specific recommendations. ^19^ To date, no phase III randomized trial has ever been conducted specifically for ChRCC. These limitations highlight the need for multi-institutional registries and dedicated prospective studies in order to better characterize pathologic and molecular predictors of aggressive ChRCC and to eventually guide management strategies.

## 4 Conclusion

This case highlights a rare and understudied case of aggressive ChRCC. While the patient's tumor demonstrated histopathological, gene expression, immunological features consistent with prior reports in the literature, it also exhibited distinct molecular characteristics that differentiate it from other documented cases of aggressive ChRCC. Surgical resection remains the standard of care; however, the rarity of aggressive ChRCC has limited the development of a broad, evidence-based consensus on optimal management strategies. Further investigation into the molecular origins and pathogenesis of this subtype is essential to inform targeted therapeutic approaches, particularly for aggressive disease presentations.

## CRediT authorship contribution statement

**Sam Kwon:** Data curation, Investigation, Methodology, Project administration, Writing – original draft, Writing – review & editing. **Tali Newman:** Data curation, Investigation, Writing – original draft. **Fiona Wardrop:** Investigation, Writing – original draft. **Katherine Zhong:** Investigation, Writing – original draft. **Josiah Dallmer:** Investigation, Writing – original draft. **Andrew Hall:** Formal analysis, Investigation, Visualization, Writing – original draft. **Michael Whalen:** Conceptualization, Methodology, Project administration, Supervision, Validation, Writing – original draft, Writing – review & editing.
